# Crystal Structure of the High-Pressure Phase of Ca(BH_4_)_2_ and Further Structural Changes up to 70 GPa

**DOI:** 10.1021/acs.inorgchem.6c00382

**Published:** 2026-03-30

**Authors:** Satoshi Nakano, Hiroshi Fujihisa, Hiroshi Yamawaki, Yuki Shibazaki, Takumi Kikegawa, Shin-ichi Orimo

**Affiliations:** † National Institute for Materials Science (NIMS), Tsukuba, Ibaraki 305-0044, Japan; ‡ National Metrology Institute of Japan (NMIJ), National Institute of Advanced Industrial Science and Technology (AIST), Tsukuba, Ibaraki 305-8565, Japan; § Photon Factory (PF), Institute of Materials Structure Science (IMSS), 13551 High Energy Accelerator Research Organization (KEK), Tsukuba, Ibaraki 305-0801, Japan; ∥ Advanced Institute for Materials Research (WPI-AIMR), 13101 Tohoku University, Katahira 2-1-1, Aoba-ku, Sendai 980-8577, Japan; ⊥ Institute for Materials Research (IMR), Tohoku University, Katahira 2-1-1, Aoba-ku, Sendai 980-8577, Japan

## Abstract

High-pressure (HP) structural changes in calcium borohydride, Ca­(BH_4_)_2_, were investigated up to 70 GPa using X-ray diffraction (XRD) measurements and Raman scattering spectroscopy. At 2.1 GPa, the ambient pressure (AP) phase (α-Ca­(BH_4_)_2_) underwent a pressure-induced phase transition to an HP phase (HP1). Rietveld refinement of the XRD patterns and density functional theory calculations revealed that the crystal structure of HP1 adopts a *P*2_1_/*c* structure. This structure is close to that of the AP phase, with a volume change of only about 1.7%. Upon further compression of HP1, another pressure-induced phase transition was suggested above approximately 20 GPa; however, the structure of this new HP state (HP2) could not be determined. Raman scattering spectra also revealed the two pressure-induced phase transitions, which are consistent with the XRD results. Although Raman measurements demonstrated that the short-range structural feature was preserved in HP2, long-range ordering was not observed upon XRD measurements, suggesting that HP2 may be an amorphous-like disordered state. Among these phase transitions, the transition from the AP phase to HP1 was reversible, whereas HP2 did not revert to HP1 or the AP phase upon decompression. The sample recovered from HP2 exhibited XRD patterns and Raman spectra similar to those of γ-Ca­(BH_4_)_2_.

## Graphical abstract

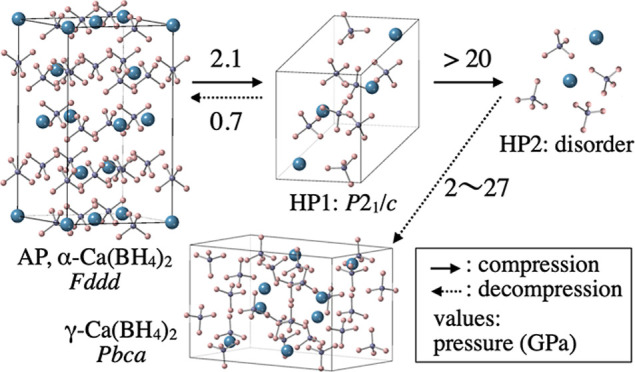

## Introduction

In recent years, the trend toward a shift from fossil fuels to hydrogen energy toward a sustainable society has led to significant advances in hydrogen science and also hydrogen-related materials. Ternary complex hydrides have long been used as reducing agents, but they are increasingly attracting attention as novel functional materials. One of these functions is their potential as hydrogen storage materials.^1^ A complex hydride, LiBH_4_, possesses large gravimetric (18.5 wt %) and volumetric (121 kg H_2_/m^3^) hydrogen densities, far exceeding United States Department of Energy (DOE) ultimate targets for automotive hydrogen storage materials (gravimetric density 6.5 wt %, volumetric density 50 kg H_2_/m^3^). Another important functional feature is the high ionic conductivity required for all-solid-state batteries. The high-temperature (HT) phase of LiBH_4_, which appears above 390 K, exhibits high ionic conductivity exceeding 2 × 10^–3^ S cm^–1^.^2,3^ Furthermore, when combined with LiNH_2_ or LiI, it has been found to maintain high Li-ion conductivity of over 1 × 10^–4^ S cm^–1^ even at room temperature (RT).^4^ However, these examples of LiBH_4_ involve the relatively scarce Li^+^ ion. Therefore, there is a strong demand for the development of functional complex hydrides based on other abundant alkali metal or alkaline earth metal ions. Ca­(BH_4_)_2_, the focus of this study, is one such candidate.^5^

We have been studying the structural changes, densification, and associated physical properties under high pressure (HP) in ternary complex hydrides. With respect to LiBH_4_, HP X-ray diffraction (XRD) measurements have revealed three pressure-induced phase transitions up to approximately 50 GPa, and the crystal structures of the three HP phases have been elucidated by density functional theory (DFT) calculations.^6^ In particular, the metastable V′ phase and the stable V phase, which appear above 17 GPa, exhibit disordered hydrogen atoms in the BH_4_^–^ complex ions, resulting in rotation of the complex ions within the crystal structure. We have reported that the V phase exhibits the second highest ionic conductivity after the HT phase due to this rotational motion of the complex ions.^7^ Such rotation of hydride ion clusters has also been reported for the HT phase transition of Na_2_B_12_H_12_.^8^ Exploring structural changes induced by HP or HT and clarifying the structural properties of polymorphic phases may lead to the discovery of new functionalities in ternary alkaline earth metal hydrides.

The polymorphism of Ca­(BH_4_)_2_ has been investigated at HT and HP. Noritake et al. clarified the crystal structures of three polymorphs of Ca­(BH_4_)_2_, namely, the α-, β-, and γ-phases, and reported that the α-Ca­(BH_4_)_2_ undergoes a phase transformation to the β-Ca­(BH_4_)_2_ at 433 K and decomposes into CaH_2_, CaB_6_, and H_2_ at 620 K.^9^ Borgschulte et al. calculated the temperature dependence of the free energies of these three phases and showed that the β-Ca­(BH_4_)_2_ is the HT phase, whereas the γ-phase is metastable in the temperature range of 0–600 K.^10^

Several HP experiments on Ca­(BH_4_)_2_ have been reported. George et al. performed HP XRD (∼14 GPa) and HP Raman scattering (∼25 GPa) measurements on a mixed sample of the α- and β-Ca­(BH_4_)_2_.^11^ They reported that α-Ca­(BH_4_)_2_ was stable up to 14 GPa, whereas the β-Ca­(BH_4_)_2_ underwent a transition to a disordered phase at 10.2 GPa, although the structure was unclear. Liu et al. performed HP Raman scattering and HP infrared measurements of the α-Ca­(BH_4_)_2_ using KBr as a pressure medium up to 10.4 GPa and reported three pressure-induced phase transitions.^12^ Li et al. conducted HP XRD (∼52 GPa) and HP Raman scattering (∼44 GPa) measurements on α-Ca­(BH_4_)_2_ containing a small amount of γ-Ca­(BH_4_)_2_ without a pressure medium, and reported that a pressure-induced phase transition to a *C*2/*c* structure occurred between 2.36 and 7.97 GPa.^13^

Nonetheless, several studies based on first-principles calculations have reported HP structural changes and phase transitions. Majzoub et al. reported that the α-Ca­(BH_4_)_2_ undergoes a phase transition to the β-Ca­(BH_4_)_2_ with *P*-4 symmetry at 5.3 GPa.^14^ Aeberhard et al. calculated the stable pressure ranges of the following phases at 0 K: α-phase (*Fddd*) below 3.4 GPa, γ-phase at 3.4–3.6 GPa, baddeleyite-type (*P*2_1_/*c*) at 3.6–9.7 GPa, columbite-type (*Pbcn*) at 9.7–34.0 GPa, and *Pnma* above 34.0 GPa.^15^ Di Cataldo et al. reported that *Fddd* undergoes successive phase transitions to *Pbca* at 2 GPa, *P*2_1_/*c* at 5 GPa, and *P*2_1_ at 8.8 GPa, and then becomes unstable at 65 GPa, incorporating hydrogen to form Ca­(BH_5_)_2_, or decomposing to CaBH_3_ or CaBH_5_.^16^

As described above, various experimental and computational results have been reported regarding the HP structural changes of Ca­(BH_4_)_2_, but no clear consensus has been reached. In experimental studies, the starting sample often consists of a mixture of α-, β-, and γ-Ca­(BH_4_)_2_, which may complicate the identification of phase transitions. Furthermore, because Ca­(BH_4_)_2_ is highly reactive with water and alcohols, most experiments have been performed using solid pressure media such as KBr or without a pressure medium. In such cases, variations in anisotropic compression may influence the occurrence and pressure range of phase transitions. In addition, broadening of diffraction patterns and spectra may hinder the identification of the phase transitions themselves. Therefore, the use of a single-phase starting sample and experiments employing hydrostatic pressure media such as helium are required to clarify the fundamental structural changes of Ca­(BH_4_)_2_ more reliably.

Hydrides have recently attracted attention as new superconductors exhibiting superconducting transition temperatures (Tc) comparable to RT under HP. H_3_S has been reported to exhibit a Tc of 203 K at 155 GPa,^17^ and LaH_10_ has been reported to exhibit a Tc of 250 K at 170 GPa.^18,19^ The search for new superconductors has thus expanded from binary to ternary hydrides. The Ca–B–H ternary hydrides have also been investigated as candidates for such superconductors, with Ca­(BH_4_)_2_ considered a potential starting material. Di Cataldo et al. calculated that Ca­(BH_4_)_2_ becomes unstable above 65 GPa, and predicted that the decomposition products CaBH_6_ and Ca_2_B_2_H_13_, derived from Ca­(BH_4_)_2_, would exhibit superconducting properties with Tc values of 119 and 89 K, respectively, at 300 GPa.^16^ In addition, Yang et al. calculated the Tc under HP for the pseudobinary system CaB–H_*n*_ (*n* = 2–11) in various space groups and predicted that the *Imm*2-type CaBH_6_ would exhibit superconducting properties with a Tc of approximately 200 K at 300 GPa.^20^

For these reasons, structural changes and physical properties of Ca­(BH_4_)_2_ under HP are currently of great interest, both with respect to Ca­(BH_4_)_2_ and as a precursor for new ternary hydrides. In this study, we investigated the pressure-induced structural changes of α-Ca­(BH_4_)_2_ up to approximately 70 GPa using XRD and Raman scattering. Helium, which provides a highly hydrostatic condition, and hydrogen, which can also serve as a hydrogen source, were used as pressure media. Furthermore, for the HP phase, for which several structures have been proposed, the most stable structure was determined using DFT calculations and molecular dynamics simulations based on the XRD patterns.

## Experimental Section

### Sample and Pressure Medium

The sample used was commercially available Ca­(BH_4_)_2_ (purity >96.5%, Sigma-Aldrich, USA). Powder XRD analysis revealed that the sample consisted almost entirely of α-Ca­(BH_4_)_2_. However, in some samples taken from the bottle, a weak diffraction peak corresponding to the strongest reflection of β-Ca­(BH_4_)_2_ was occasionally observed. Samples containing detectable β-Ca­(BH_4_)_2_ were generally not used in the experiments. All sample handling was performed in an argon glovebox with oxygen and water vapor concentrations of 1 ppm or less.

Helium gas (G1 grade, purity: 99.99995%) and hydrogen gas (G1 grade, purity: 99.99999%) were prepared as pressure media. In this study, helium, which provides the highest degree of hydrostaticity, was primarily used as the pressure medium. In some experiments, hydrogen was used to examine the possibility that Ca­(BH_4_)_2_ decomposes to form compounds requiring additional hydrogen.

### Preparation of the HP Cells

Diamond-anvil-cells (DACs) equipped with a pair of 1/4-carat diamond anvils with 300–600 μm diameter culets were used for XRD and Raman scattering measurements. Most experiments were performed at RT and under HP using a rectangular prism-type DAC with external dimensions of 50 mm × 50 mm × 55 mm. A rhenium foil, 250–300 μm thick, was pre-indented between the anvils to a thickness of 60–70 μm and used as a gasket. A 150–300 μm diameter hole was drilled at the center of the gasket using an electrical discharge machine, forming the sample chamber together with the anvils. The sample powder and ruby balls (10–15 μm in diameter), used as pressure markers,^21,22^ were loaded into the sample chamber. The remaining volume of the sample chamber was filled with the pressure medium (helium or hydrogen) using a gas-loading system.^23^

For annealing experiments, a Mao–Bell DAC was combined with a band heater to perform HP and HT experiments using external heating. Details of the external heating procedure have been described in a previous report.^24^ For pressure measurements at HT, Sm^2+^-doped yttrium-aluminum garnet (SrB_4_O_7_:Sm^2+^) powder was placed in the sample chamber instead of ruby balls, and the pressure was estimated from the shift of the fluorescence line (λ1).^25^ The λ1 fluorescence line of SrB_4_O_7_:Sm^2+^ retains relatively strong intensity compared to ruby fluorescence at HT, and its peak shift is temperature-independent, making it suitable for pressure measurements under HT conditions.

### HP XRD Measurements

Angle-dispersive XRD measurements were conducted using synchrotron radiation at the BL-18C and AR-NE1A beamlines of the Photon Factory at the High Energy Accelerator Research Organization (KEK-PF). The X-ray beam was monochromatized to 20 keV at BL-18C and 30 keV at AR-NE1A and introduced to the sample in the DAC through a collimator with a 30–100 μm diameter pinhole. Two-dimensional (2D) diffraction patterns were collected in transmission geometry using flat-panel detectors (Teledyne Rad-Icon Imaging Corp., Rad-icon 2022 and Rad-icon 1520) for room-temperature experiments and an image plate for HT experiments. Pressure was increased up to approximately 70 GPa and the X-ray exposure time was approximately 15 min. The 2D diffraction patterns were integrated along the radial direction into one-dimensional (1D) profiles using the image analysis software, IPAnalyzer.^26^ Lattice parameters and unit cell volumes were calculated using PDIndexer.^26^ The Rietveld analyses of the powder XRD patterns were performed using BIOVIA Materials Studio (MS) Reflex, version 2024 SP1.^27^

The bulk modulus of each phase was calculated from the equation of state (EoS). The EoS was obtained by fitting a third-order Birch–Murnaghan equation^28^ using the fitting calculation software EoSFit7c.^29^

### HP Raman Scattering Measurements

A continuous-wave fiber laser with a wavelength of 488 nm (Azur Light Systems, ALS-BL-488-1-I-SF, 1 W) was used as the excitation source for the Raman scattering experiments, with 20 mW of power delivered to the sample surface. The incident laser beam was focused to a diameter of approximately 10 μm on the sample through the diamond anvil window using a 45× objective lens. Raman scattering spectra were collected using a single-monochromator spectrometer (Jobin-Yvon/Atago-Bussan T64000) equipped with a 600-grooves/mm diffraction grating, providing a spectral dispersion of 2 cm^–1^ per pixel. The spectra were recorded for durations of 40–100 s using a silicon-based charge coupled device (Spectrum ONE, ISA Inc.) with 2000 × 800 pixels, electrically cooled to 140 K. The wavenumber scale was calibrated using a neon lamp.

### Computational Structural Analysis

Structural information, such as the coordinates of hydrogen atoms, could not be obtained from the XRD experiments. Therefore, full atomic structure optimization was performed using MS-CASTEP^30^ while fixing the lattice constants to those obtained from the Rietveld analysis.

The Perdew–Burke–Ernzerhof exchange–correlation functional within the generalized gradient approximation framework^31^ and on-the-fly generated norm-conserving pseudopotentials^32^ were employed. A plane-wave basis set with a cutoff energy of 570.0 eV was used. For the primitive cell of the α-phase, a 3 × 3 × 3 Monkhorst–Pack (MP) grid^33^ was selected for *k*-point sampling, resulting in *k*-point separations of approximately 0.07 Å^–1^. For the monoclinic cell of the first HP phase, a 2 × 2 × 2 MP grid^33^ was employed. The convergence criteria included a maximum force tolerance of 0.01 eV/Å, a maximum atomic displacement of 5.0 × 10^–4^ Å, and an energy convergence tolerance of 5.0 × 10^–6^ eV/atom.

## Results and Discussion

### Pressure-Induced Phase Transition to the First HP Phase, HP1

The change in the XRD pattern during compression up to approximately 28 GPa is shown in Figure 1. All diffraction peaks at 0.4 GPa, near ambient pressure, were assigned to α-Ca­(BH_4_)_2_ (Figure S1 in the Supporting Information). This phase is hereafter referred to as the AP phase. In previous reports, the *Fddd* model^5^ and the *F*2*dd* model^34^ have been used to describe the crystal structure of this AP phase. However, structural optimization using DFT calculations in this study resulted in an automatic transformation from *F*2*dd* to *Fddd*, indicating that the *Fddd* model is more stable. Therefore, the crystal structure model for the AP phase is referred to as *Fddd*. The main difference between the *Fddd* and *F*2*dd* models lies in the orientation of the BH_4_^–^ complex ions.

**Figure 1:**
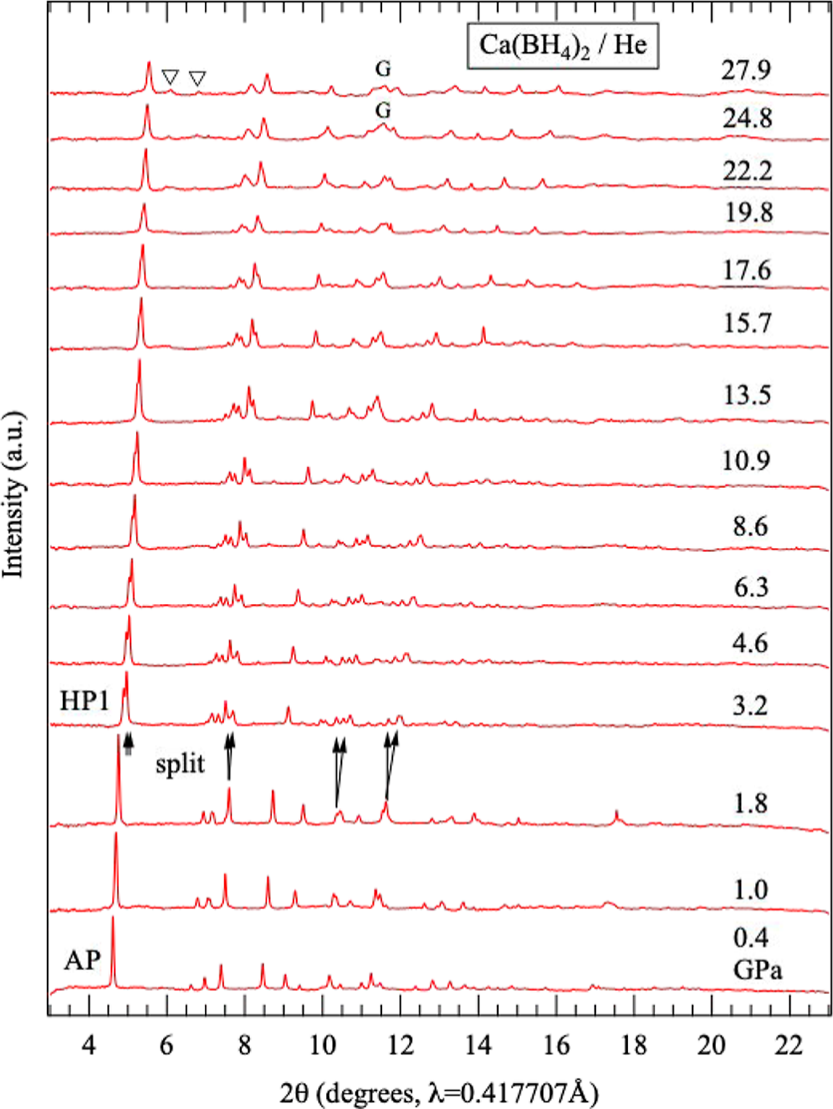
Pressure dependence of the XRD patterns of α-Ca­(BH_4_)_2_ up to approximately 30 GPa. The arrows indicate peak splitting associated with the pressure-induced phase transition from the AP phase to HP1. “G” denotes diffraction peaks from the gasket, and the inverted triangle indicates a weak, broad peak that appears above 20 GPa.

Upon compression of the AP phase, splitting of several XRD peaks was observed between 1.8 and 3.2 GPa, suggesting a pressure-induced phase transition. Detailed measurements revealed that this phase transition occurred at approximately 2.1 GPa. The HP phase appearing after this transition is designated HP1. With further compression, the split peaks gradually converged and eventually merged back into single peaks.

Upon decompression, HP1 reverted to the AP phase at approximately 0.7 GPa. This reversible transformation occurred whether the pressure was released from approximately 3 GPa, immediately after the AP-to-HP1 phase transition, or from approximately 20 GPa. In other words, the pressure-induced phase transition between the AP phase and HP1 is a reversible, although it is accompanied by hysteresis.

When HP1 was further compressed above 20 GPa, several weak peaks appeared around 2θ = 6–8° in Figure 1. This observation suggests the occurrence of an additional pressure-induced phase transition, which is discussed in detail below.

### Crystal Structure of HP1

The crystal structure of HP1 observed at pressures between 2.1 and 20 GPa was determined by Rietveld refinement of the experimental XRD patterns and combined with DFT calculations. The structural analysis described below revealed that HP1 adopts a *P*2_1_/*c* structure. The Rietveld refinement results and the corresponding crystal structure model of HP1 are shown in Figure 2.

**Figure 2:**
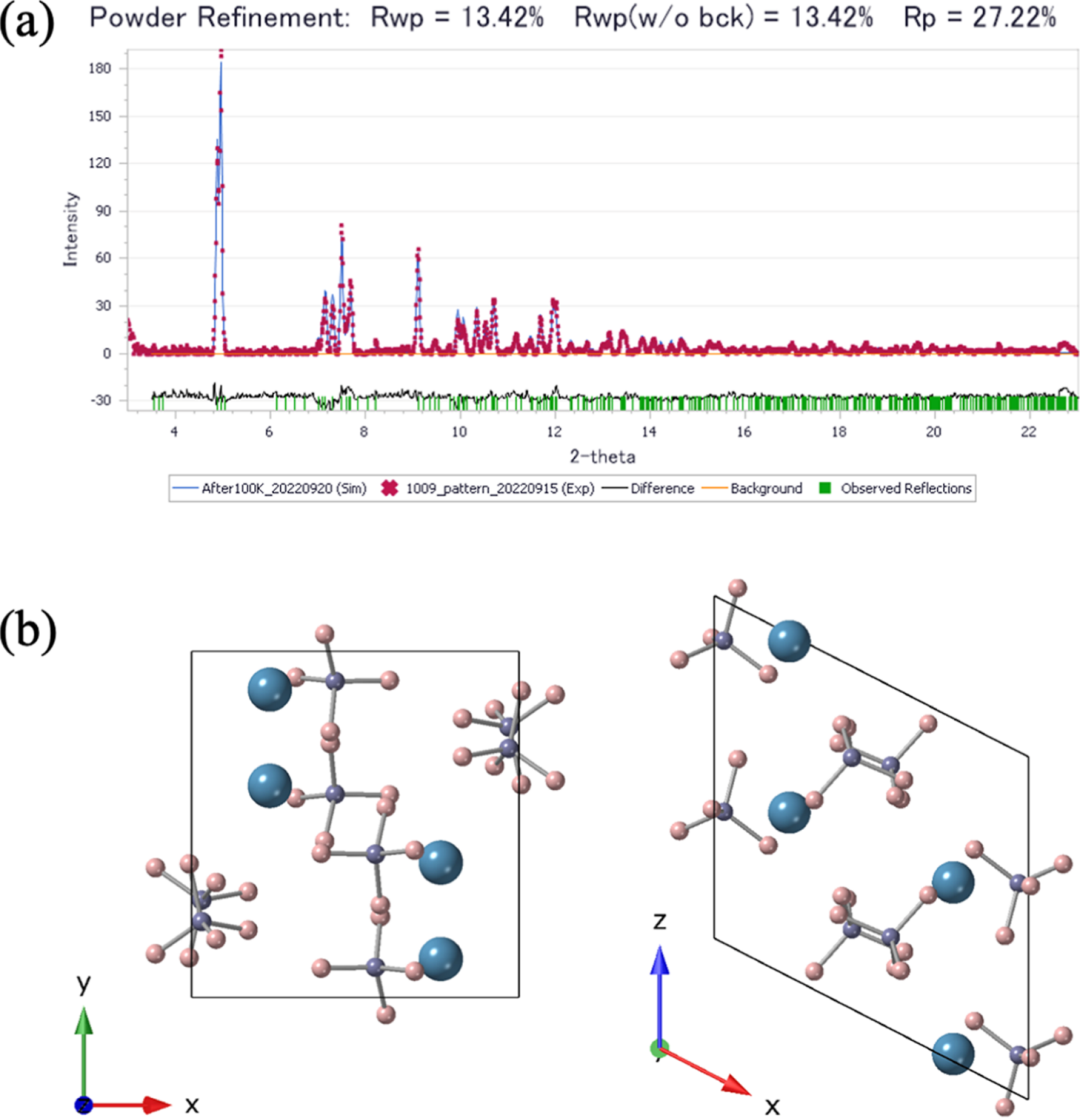
Result of the Rietveld refinement and structural models of HP1. (a) Rietveld refinement of the XRD pattern obtained at 3.2 GPa and RT based on the *P*2_1_/*c* structure. Red dots and blue lines represent the observed and calculated intensities, respectively. Vertical green bars indicate the positions of the calculated diffraction lines. Black line at the bottom shows the difference between the observed and calculated intensities. (b) Crystal structure of HP1 refined in the *P*2_1_/*c* space group. The left and right panels show the *ab* and *ca* planes, respectively. Blue, purple, and pink spheres represent calcium, boron, and hydrogen atoms, respectively.

Rietveld refinement was carried out with reference to previously proposed structural models. The *C*2/*c* model (*Z* = 4) reported by Li et al.^13^ and the *P*2_1_/*c* model reported by Aeberhard et al.^15^ yielded lower *R*_wp_ values than other models and were therefore identified as the most plausible structural candidates. DFT calculations for both models showed that the enthalpy at 3 GPa was −5271.75632 eV for the former and −5271.78565 eV for the latter, indicating that the *P*2_1_/*c* model is more stable. Although zero-point energy and finite-temperature vibrational effects may slightly modify the relative stability, the two structures are very similar, and the relatively large energy difference of approximately 30 meV suggests that the phase stability ordering is likely robust. In addition, optimization of the lattice parameters revealed that the *C*2/*c* model failed to reproduce the experimental XRD pattern, whereas the *P*2_1_/*c* model provided a good fit. Based on these results, the crystal structure of HP1 is concluded to be the *P*2_1_/*c* model.

With respect LiBH_4_, we previously reported that hydrogen disordering and rotation of BH_4_^–^ complex ions occurred in the HP phase of LiBH_4_ (phase V).^6^ We also demonstrated that, as a result of this rotational motion of the complex ions, the HP phase of LiBH_4_ exhibits the second highest ionic conductivity after the HT phase (phase I).^7^ Molecular dynamics simulations were performed for Ca­(BH_4_)_2_ HP1 to examine whether similar behavior occurs; however, no hydrogen disordering was observed, and the BH_4_^–^ complex ions were found to remain stationary within the crystal structure.

The relationship between the crystal structures of the AP phase and HP1 is illustrated in Figure 3. The unit cell of HP1 (*P*2_1_/*c*) is a parallelogram in the *ac* plane, with its two diagonals corresponding to the *a*- and *b*-axes of the AP unit cell (*Fddd*). The *b*-axis of the *P*2_1_/*c* structure is the shortest axis, and corresponds to the *c*-axis of the *Fddd* structure. During the phase transition from the AP phase to HP1, structural changes occur mainly through slight displacements and rotations of adjacent BH_4_^–^ complex ions, allowing the available space between ions to be more efficiently filled. Concurrently, the arrangement of Ca^2+^ ions changes from a flat configuration to a slightly wavy one. Nevertheless, the overall structures of the AP phase and HP1 remain very similar, which is considered to be responsible for the smooth and reversible nature of the phase transition between them.

**Figure 3:**
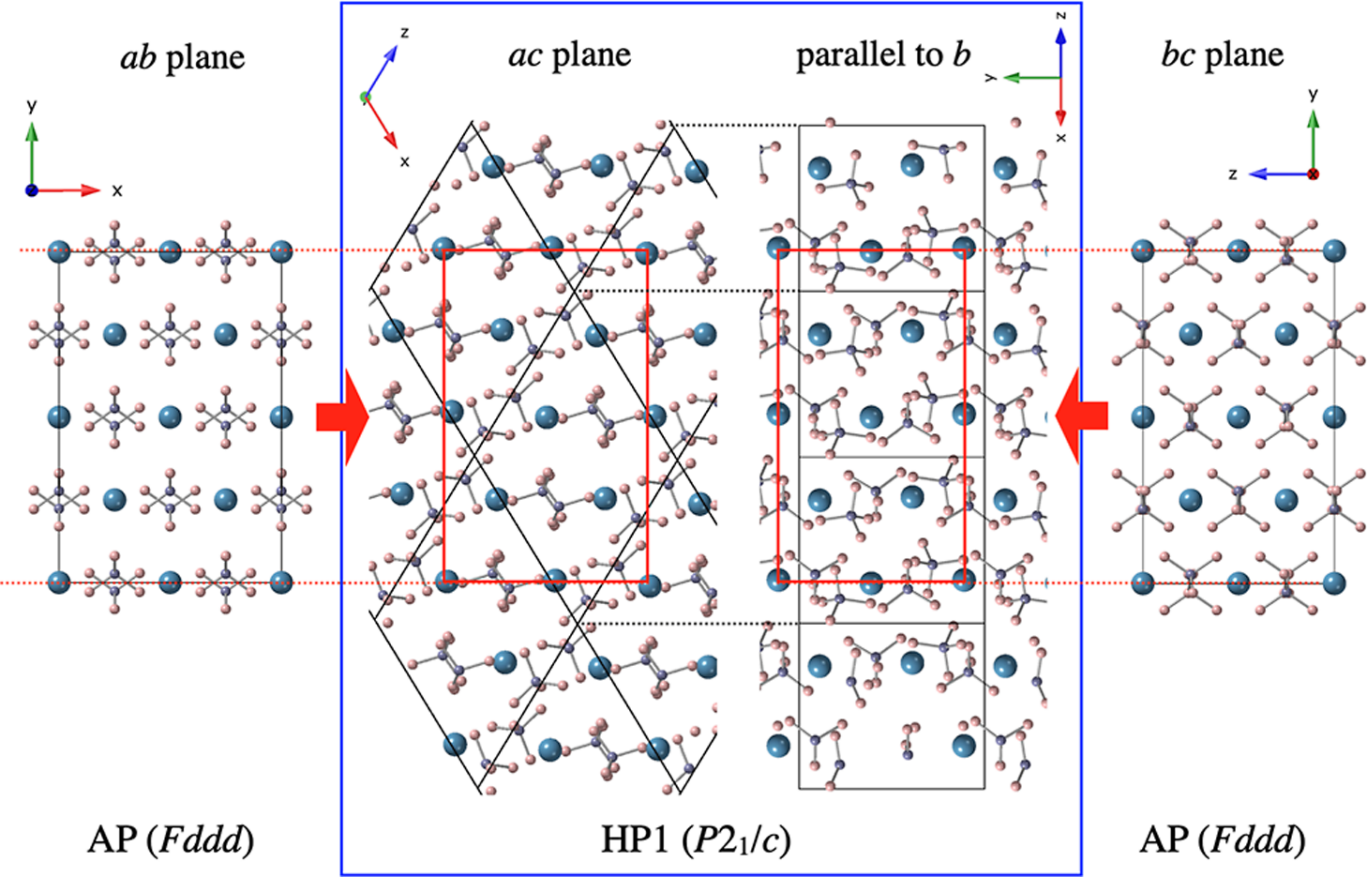
Relationship between the crystal structures of the AP phase and HP1. Blue, purple, and pink spheres represent calcium, boron, and hydrogen atoms, respectively.

### Pressure Dependence of Volume

Using the structural model obtained in this study, the pressure dependence of the volumes of the AP phase and HP1 was calculated (Figure 4). The volume decrease associated with the phase transition from the AP phase to HP1 was only 1.7%, reflecting the small structural change accompanying this transition.

**Figure 4:**
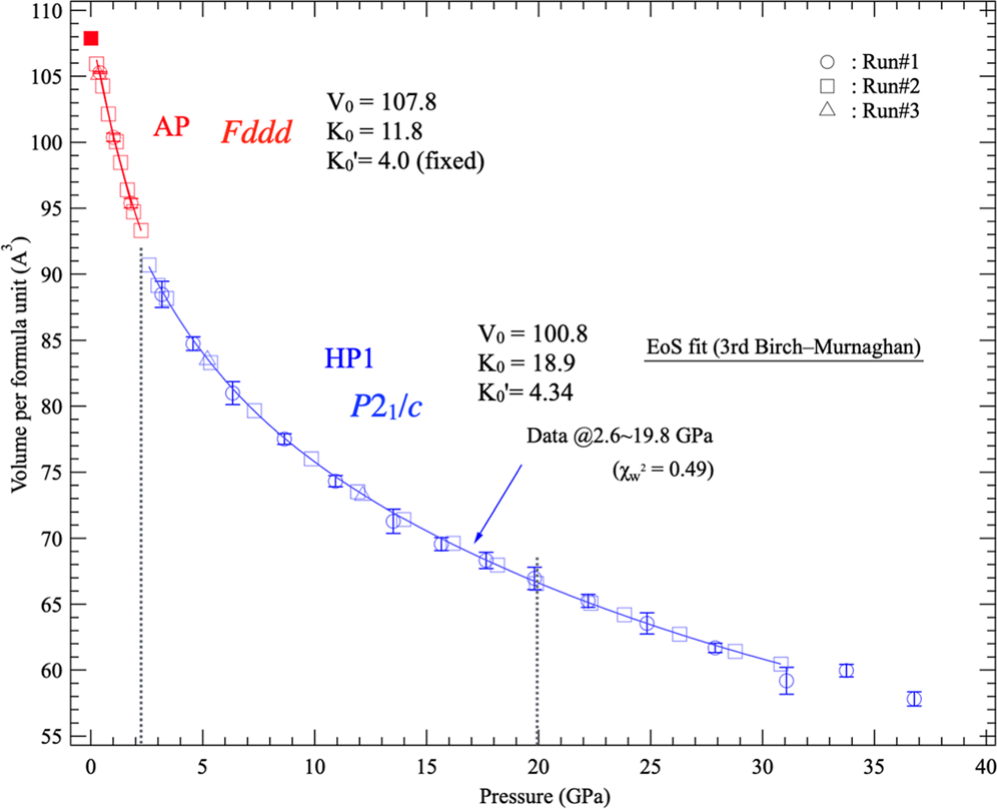
Pressure dependence of the formula-unit volumes of the AP phase and HP1. The blue solid line represents the equation of state (EoS) fitted using data up to 20 GPa, where only HP1 peaks are observed.

The EoS curve for HP1 was obtained by fitting a third-order Birch–Murnaghan equation using data up to 20 GPa, where only HP1 diffraction peaks were observed. The weighted chi-squared values, which indicate the quality of the fit, were below 1 in both cases and were nearly identical.

The bulk modulus of the AP phase was calculated to be *K*_0_ = 11.8 GPa (*V*_0_ = 107.8 Å^3^, *K*_0_′ was fixed at 4.0 due to the small number of data points). For HP1, fitting using data up to 20 GPa yielded *K*_0_ = 18.9 GPa (*V*_0_ = 100.8 Å^3^, *K*_0_′ = 4.34).

In XRD experiments reported by George et al.,^11^ no pressure-induced structural changes were observed for α-Ca­(BH_4_)_2_ up to approximately 14 GPa. Li et al.^13^ reported a pressure-induced phase transition at around 2.36–7.97 GPa, and calculated bulk moduli of 19.5 GPa for the ambient-pressure phase and 28.9 GPa for the HP phase. The discrepancy between those results and the present study is attributed to differences in the pressure media, and hence differences in hydrostaticity. Because neither George et al. nor Li et al. used a pressure medium, the splitting of XRD peaks that was clearly observed in the present experiments was not detected, suggesting that the phase transition pressure and the structural analysis of the HP phase were not fully resolved. In addition, in the bulk modulus calculation by Li et al., the compression curve deviated from the experimental data points. The results of the present study are therefore considered to more accurately reflect the intrinsic behavior of α-Ca­(BH_4_)_2_ under HP.

Aeberhard et al.^15^ evaluated the volume dependence of the enthalpy for several Ca­(BH_4_)_2_ structures using first-principles calculations. Applying their results to the volumes obtained in this study, phase transitions were predicted from the *Fddd* structure of α-Ca­(BH_4_)_2_ to the baddeleyite-type *P*2_1_/*c* structure at approximately 2 GPa, to the columbite-type *Pbcn* structure at 9 GPa, and to the cotunnite-type *Pnma* structure at 21 GPa. The predicted baddeleyite-type *P*2_1_/*c* structure is consistent with the present experimental results, whereas the transition to the columbite-type *Pbcn* structure was not observed in this study.

### New Diffraction Peaks Appearing Above 20 GPa

As shown in Figure 1, above 20 GPa, several new weak peaks that could not be assigned to HP1 appeared at 2θ = 6.0°, 6.7°, and 7.3°, corresponding to interplanar spacings of *d* = 4.0, 3.6, and 3.3 Å, respectively. Figure 5 shows the evolution of the XRD patterns obtained in experiments extended to approximately 70 GPa. With increasing pressure, the HP1 peaks weakened, whereas the newly emerged peaks became more distinct. This behavior suggests that a new structural change from HP1 occurs above 20 GPa. This state observed above 20 GPa is tentatively referred to as HP2. These new peaks were reproducible in separate measurements; however, they were consistently weak and broad, and their relative intensities varied between experimental runs. In addition to these peaks, several broader features were observed at higher 2θ angles. These broad peaks could not be reliably indexed, making it impossible to determine whether they originated from a single phase. Consequently, crystal structure analysis of HP2 was not feasible. Aeberhard et al.^15^ predicted that the cotunnite-type *Pnma* structure becomes stable above approximately 21 GPa; however, the newly observed HP2 peaks could not be indexed using the *Pnma* model.

**Figure 5:**
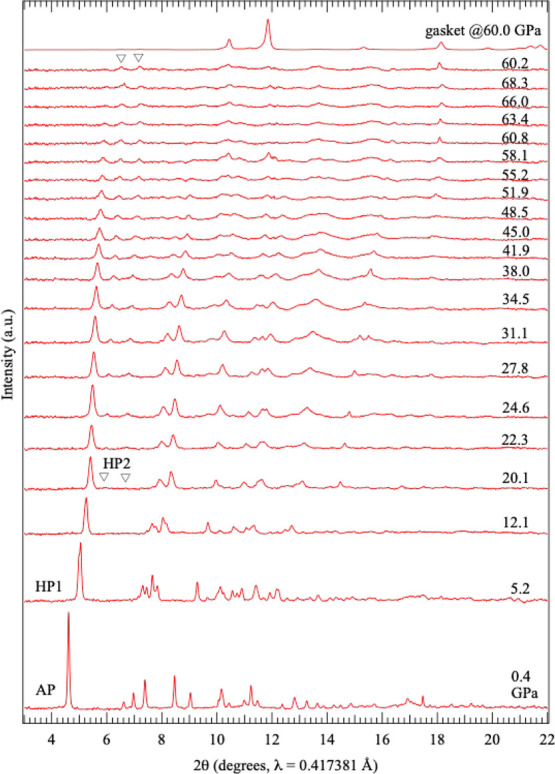
Pressure dependence of the XRD patterns of α-Ca­(BH_4_)_2_ up to approximately 70 GPa. After measurements at 68.3 GPa, the pressure was reduced to 60.2 GPa after approximately 12 h. The top image shows the XRD pattern of the Re gasket.

Di Cataldo et al.^16^ investigated the structural stability of Ca­(BH_4_)_2_ using first-principles calculations and claimed that Ca­(BH_4_)_2_ becomes unstable above 65 GPa. The weak peaks observed above 20 GPa in the present study may therefore not represent a new HP phase of Ca­(BH_4_)_2_, but instead decomposition or reaction products. Although Di Cataldo et al. proposed possible compounds and structural models for the decomposition of Ca­(BH_4_)_2_, comparison with these models did not identify any structure consistent with the HP2 peaks observed in this study (Figure S2). If Ca­(BH_4_)_2_ decomposes above 20 GPa, the presence of excess hydrogen surrounding Ca­(BH_4_)_2_ could lead to different reaction products. To examine this possibility, additional experiments were performed using hydrogen instead of helium as the pressure medium. However, the XRD patterns obtained up to 70 GPa were essentially identical to those measured using helium as the pressure medium.

### Diffraction Pattern of Samples Recovered from Pressures Above 20 GPa

Upon decompression of HP2, no reverse transition to HP1 or the AP phase was observed, and the characteristic peaks described above were retained at least down to approximately 2.7 GPa. Further decompression led to changes in the diffraction pattern near ambient pressure (Figure S4). Therefore, based solely on the evolution of the diffraction patterns during decompression, it was not possible to determine whether the peaks observed for HP2 correspond to a new HP phase of Ca­(BH_4_)_2_ or to decomposition products.

Figure 6 shows a representative diffraction pattern of a sample recovered from HP2 near ambient pressure. The diffraction pattern of the recovered sample clearly differs from that of the starting α-Ca­(BH_4_)_2_, and more closely resembles that of γ-Ca­(BH_4_)_2_. If this phase represents the primary product transformed from HP2, it may suggest that HP2 is not a decomposition product but rather a new HP phase of Ca­(BH_4_)_2_. Furthermore, it is possible that decompression induced a phase transition from HP2 to γ-Ca­(BH_4_)_2_, instead of a reverse transition back to HP1 and then to the AP phase (α-Ca­(BH_4_)_2_).

**Figure 6:**
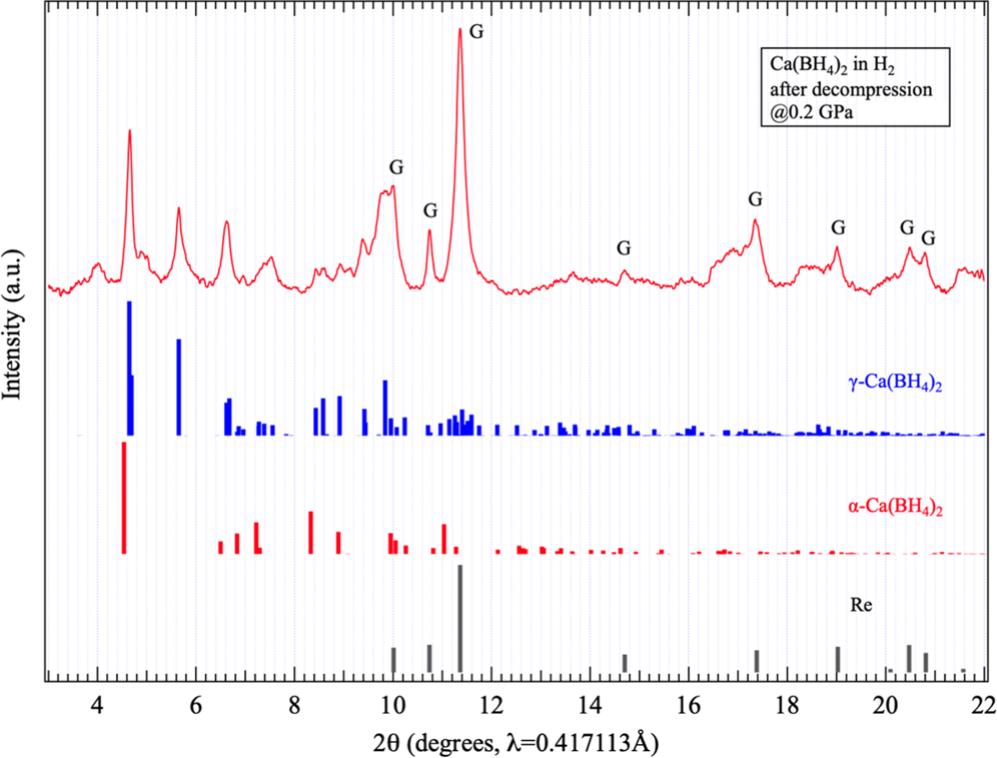
XRD pattern of a sample pressurized to 64.3 GPa in hydrogen and subsequently decompressed to 0.2 GPa. A similar pattern was also obtained using helium as the pressure medium. “G” indicates the gasket peak. Black, red, and blue vertical lines represent simulated diffraction peaks at ambient pressure for Re, α-Ca­(BH_4_)_2_, and γ-Ca­(BH_4_)_2_, respectively.

A comparison of the crystal structures of the α-Ca­(BH_4_)_2_ and γ-Ca­(BH_4_)_2_ is shown in Figure S5. Although the ionic arrangements of α-Ca­(BH_4_)_2_ and HP1 are very similar (Figure 3), the ionic arrangement of γ-Ca­(BH_4_)_2_ is significantly different from them. Therefore, the phase transition between α-Ca­(BH_4_)_2_ and HP1 is expected to involve only a small amount of ion movement, whereas the phase transition between α-Ca­(BH_4_)_2_ or HP1 and γ-Ca­(BH_4_)_2_ may require a much larger amount of ion diffusion.

Aeberhard et al.^15^ reported that γ-Ca­(BH_4_)_2_ is slightly more stable than α-Ca­(BH_4_)_2_ at higher pressures, although the energy difference between the two phases is very small. In such a case, a direct transformation from α-Ca­(BH_4_)_2_ to γ-Ca­(BH_4_)_2_ may be kinetically hindered during compression, whereas γ-Ca­(BH_4_)_2_ may form upon decompression without reverting to α-Ca­(BH_4_)_2_. A similar phenomenon has been reported for the structural phase transitions of TiO_2_. TiO_2_ exhibits denser phases in the sequence rutile (tetragonal, space group: *P*4_2_/*mnm*) → columbite (orthorhombic, *Pbcn*) → baddeleyite (monoclinic, *P*2_1_/*c*). However, the rutile-to-columbite transition is highly sluggish; when rutile is compressed at RT, it transforms directly to the baddeleyite phase above 12 GPa without passing through the columbite phase. Upon decompression, the baddeleyite phase transforms to the columbite phase at approximately 8 GPa.^35,36^ In the present study, HP2 did not revert to α-Ca­(BH_4_)_2_ upon decompression, and a diffraction pattern similar to that of γ-Ca­(BH_4_)_2_ was obtained. This behavior can be regarded as analogous to the TiO_2_ case. Aeberhard et al.^15^ pointed out the structural similarity between Ca­(BH_4_)_2_ and TiO_2_ and investigated some structural models for the undiscovered HP phases of Ca­(BH_4_)_2_. In this sense, it is interesting that similarities in the structural phase transitions upon decompression were observed between Ca­(BH_4_)_2_ and TiO_2_ in the present study.

Nevertheless, these observations alone do not conclusively demonstrate that HP2 is a new HP phase of Ca­(BH_4_)_2_. Further clarification may be achieved in future studies by investigating pressure-induced structural changes using γ-Ca­(BH_4_)_2_ as the starting material.

### Pressure Dependence of Raman Scattering Spectrum

Because XRD did not provide diffraction patterns sufficient for crystal structure analysis of HP2, Raman scattering spectroscopy was employed to investigate HP structural changes in the local structure. α-Ca­(BH_4_)_2_ was compressed to approximately 61 GPa in a helium pressure medium, and the Raman scattering spectra were measured.

The Raman spectrum at 0.3 GPa after loading the sample and helium gas is shown in Figure S6, together with results of simulations based on DFT calculations. The pressure dependence of the Raman spectra during compression is shown in Figure 7a,b, and that during decompression is shown in Figure 7c.

**Figure 7:**
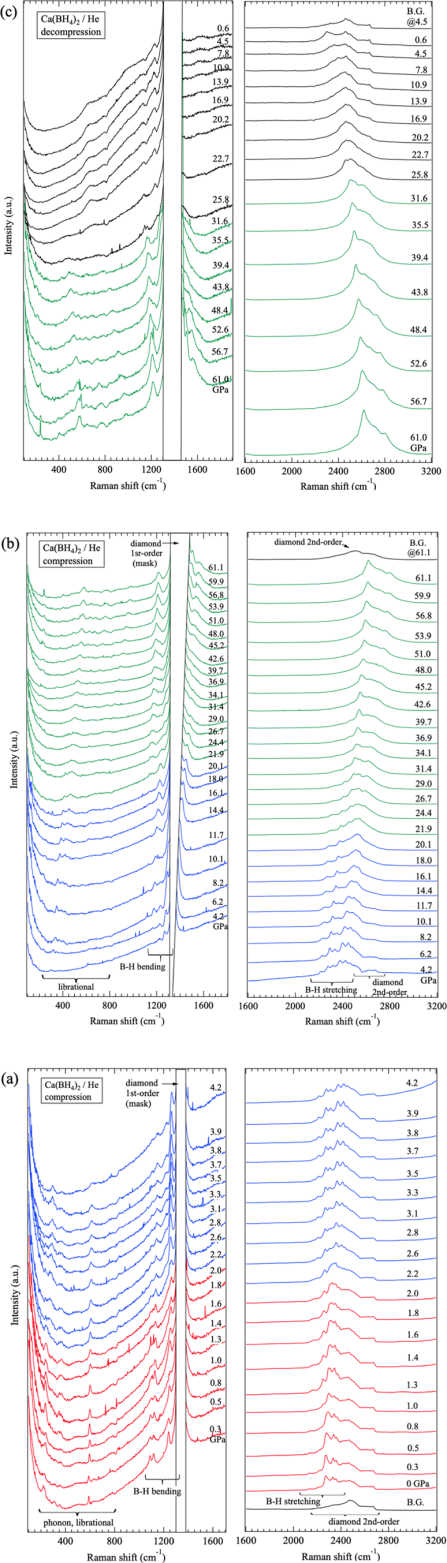
Pressure dependence of the Raman spectra of α-Ca­(BH_4_)_2_ during (a) compression from 0.3 to 4.2 GPa, (b) compression from 4.2 to 61.1 GPa, and (c) decompression from 61.1 to 0.6 GPa. Red, blue and green spectra correspond to the AP phase, HP1 and HP2 of Ca­(BH_4_)_2_.

With increasing pressure, changes in the wavenumbers and the number of Raman peaks were observed between 2.0 and 2.6 GPa for the lattice and librational modes (<600 cm^–1^), B–H bending modes (100–1500 cm^–1^), and B–H stretching modes (2000–2600 cm^–1^). These changes correspond to the pressure-induced phase transition from the AP phase (*Fddd*) to HP1 (*P*2_1_/*c*) observed by XRD. Within the pressure range of 2.1–20 GPa, identified as the HP1 region based on the XRD results, a difference in the B–H stretching vibration spectrum appears around 10 GPa. Therefore, Raman spectra at 3.2 and 16 GPa were simulated using the *P*2_1_/*c* model. The experimental spectra and the simulated results showed very good agreement at both pressures (Figure S7). Thus, Raman scattering measurements also confirmed the stable existence of HP1 in the pressure range of 2.1–20 GPa.

Above 20 GPa, a pronounced change in the Raman spectra was observed, consistent with the XRD results indicating a structural transition from HP1 to HP2. Even above 20 GPa, the B–H bending and B–H stretching modes were clearly observed, and their intensities did not decrease. However, the B–H bending modes of HP2 exhibited a discontinuous shift to lower wavenumbers compared with those of HP1. In contrast, the pressure dependence of the B–H stretching modes did not show such a discontinuous change, and rather continued smoothly from the pressure shift observed for HP1. This behavior suggests that B–H bonds similar to those in HP1 are preserved in HP2, although the bonding environment is altered. In other words, the B–H bonds remain intact even above 20 GPa, and a structural phase transition occurs.

The spectral features differ between the pressure ranges of 20–30 GPa and 30–60 GPa. Figure 8 shows the pressure dependence of the Raman peak positions in compression. At 20–30 GPa, many peaks persist from HP1, suggesting that the spectral features of HP1 and HP2 may overlap. This region likely corresponds to the sluggish transition process from HP1 to HP2 observed in XRD measurements. However, new Raman peaks also appear at around 30–35 GPa, indicating that an additional structural change may be occurring in the HP2 region at this pressure.

**Figure 8:**
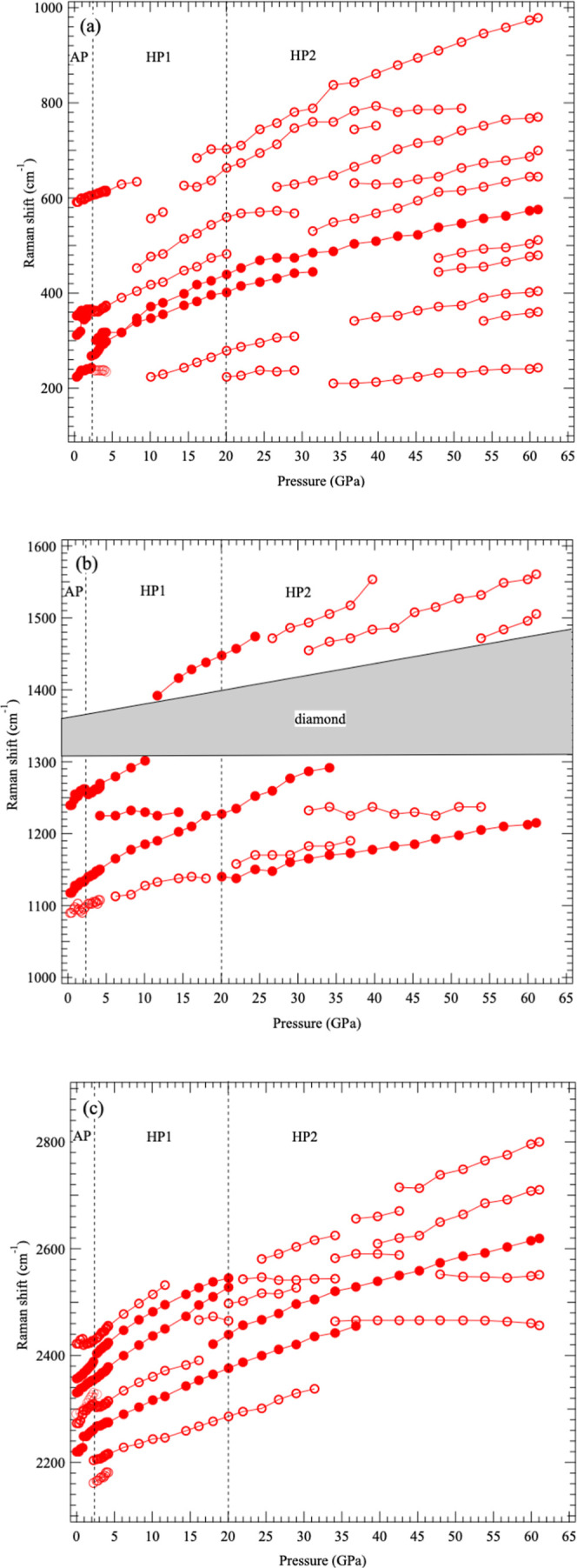
Pressure dependence of Raman peak wavenumbers in the regions of (a) 100–1000 cm^–1^, (b) 1000–1600 cm^–1^ and (c) 2100–2900 cm^–1^ during compression. Relatively strong Raman peaks are indicated by solid circles, and relatively weak peaks by open circles. The gray area around 1300–1400 cm^–1^ indicates the region obscured by the first-order peak of the diamond anvil.

At 61.1 GPa, the B–H stretching modes form a broad spectrum consisting of approximately five peaks. At the pressure, the color of HP2 was an opaque dark gray although the starting sample α-Ca­(BH_4_)_2_ was white powder.

During decompression, the Raman spectrum of HP2 changed to a different one at approximately 27 GPa (Figure 7c). At the same time, the appearance of the sample changed dramatically from opaque dark gray to colorless and translucent. These suggested the HP2 transformed to another phase at the pressure although XRD did not clearly reveal this. The spectrum of the sample recovered to near ambient pressure (0.6 GPa) is shown in Figure S8, together with the simulated spectrum of γ-Ca­(BH_4_)_2_. Although lattice and librational modes are scarcely observed in the recovered sample, the B–H bending and B–H stretching modes are similar to the simulated results for γ-Ca­(BH_4_)_2_. Both the XRD and Raman results indicated that the characteristics of the sample recovered from HP2 at near ambient pressure were similar to the γ-Ca­(BH_4_)_2_.

In addition to the wavenumber range shown in Figure 7, Raman spectra were also measured in the 3000–4800 cm^–1^ range; however, no vibrational peaks corresponding to an H_2_ vibron were observed at any pressure.

### Structure of HP2

The HP Raman scattering results demonstrate that B–H bonds are preserved even in the HP2 pressure region (*P* > 20 GPa) and that no H_2_ vibron peaks appear. Therefore, the B–H units remain intact and no molecular hydrogen was formed. It is suggested that although HP2 possesses short-range structural order, long-range order is not established possibly because of kinetic limitations, which may explain why a clear diffraction pattern was not obtained in the XRD measurements.

If HP2 possesses short-range order while long-range order is not fully developed, HT annealing under HP may promote the formation of a clearer diffraction pattern for HP2. Therefore, HP2 was annealed at 350 °C and approximately 30 GPa using an externally heated DAC. With increasing temperature, the HP1 peaks decreased in intensity; however, the HP2 peaks did not show a significant increase. Consequently, even after annealing, a diffraction pattern of HP2 with sufficient quality for structural analysis could not be obtained, and the crystal structure of HP2 could not be determined. Details of the HP/HT annealing experiment are provided in Figure S9 in the Supporting Information

As a result of comprehensive consideration of these findings, we speculate that HP2 is a relatively stable amorphous-like disordered state of Ca­(BH_4_)_2_ that lacks a long-range order. Furthermore, another structural change may occur within this disordered state around 30–35 GPa. Therefore, more detailed experiments and studies are required to reach a definitive conclusion. Meanwhile, we are interested in the physical properties of this disordered state, given that HP2 exhibits a different color to the other phases, suggesting different properties.

By combining the XRD, Raman scattering and theoretical results, pressure-induced structural changes of α-Ca­(BH_4_)_2_ during compression up to approximately 70 GPa and subsequent decompression can be summarized as shown in Figure 9.

**Figure 9:**
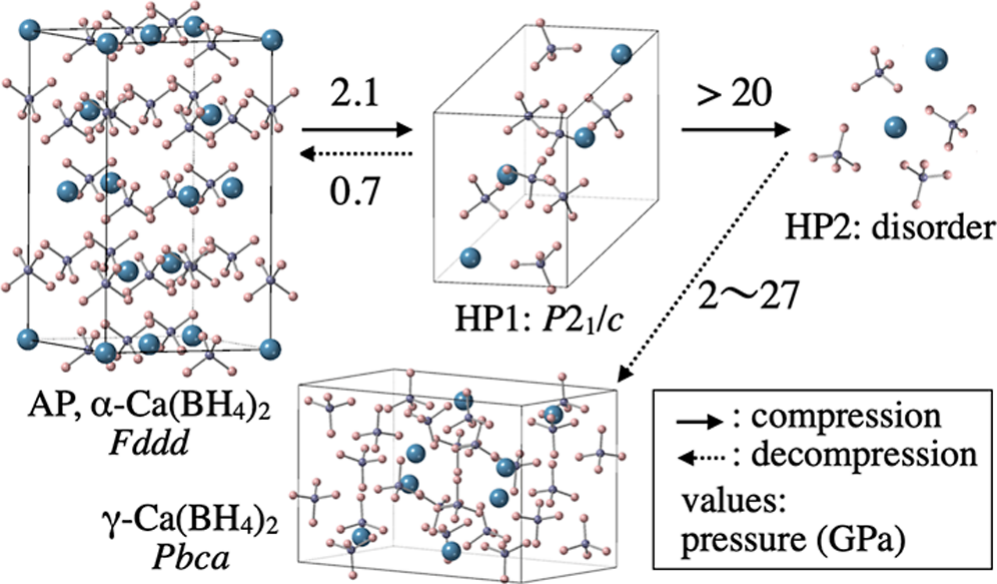
Schematic illustration of the phase changes of α-Ca­(BH_4_)_2_ during compression and decompression, based on HP XRD and Raman scattering results. Blue, purple, and pink spheres represent calcium, boron, and hydrogen atoms, respectively. The structure of HP2 was suggested to be an amorphous-like disorder, and the structural drawing in the figure is an image.

## Conclusion

To investigate the HP structural changes of α-Ca­(BH_4_)_2_, HP XRD and HP Raman scattering measurements were performed up to approximately 70 GPa. The AP phase, α-Ca­(BH_4_)_2_, was found to undergo a pressure-induced phase transition at 2.1 GPa to an HP phase (HP1) with a *P*2_1_/*c* structure. The HP1 structure is similar to that of the AP phase with a volume change of only approximately 1.7%. Because the hydrogen atoms in HP1 are ordered and the BH_4_^–^ complex ions do not undergo rotational motion, Ca^2+^ ion conduction is unlikely in HP1. Further compression indicated another pressure-induced structural change above approximately 20 GPa. However, although short-range order was present, long-range order was not established, and the structure of this new HP state (HP2) could not be determined. We speculate that HP2 is a relatively stable amorphous-like disordered state of Ca­(BH_4_)_2_. The phase transition between the AP phase and HP1 was reversible, whereas HP2 did not revert to the original structure upon decompression and instead exhibited XRD patterns and Raman spectra similar to those of γ-Ca­(BH_4_)_2_.

## Supplementary Material



## Notes

The result of Rietveld refinement and structural model of the starting sample, α-Ca­(BH_4_)_2_; comparison of the diffraction patterns of HP2 with some structural models; hydrogenation behavior of a rhenium gasket in the experiment using hydrogen as the pressure medium; changes in the XRD patterns of HP2 during decompression; comparison of the crystal structures of α-Ca­(BH_4_)_2_ and γ-Ca­(BH_4_)_2_; Raman scattering spectrum of the starting sample, α-Ca­(BH_4_)_2_; comparison of Raman scattering spectra of HP1 with simulation results; comparison of Raman scattering spectrum at the initial stage of compression with spectra recovered from HP2 at near AP; the result of HP/HT annealing of HP2 (PDF)
