# Supplementary material for: Crystal Structure of the High-Pressure Phase of Ca(BH4)2 and Further Structural Changes up to 70 GPa
Source: Inorg Chem. 2026 Mar 30;65(14):7954–64. doi: 10.1021/acs.inorgchem.6c00382 (PMC13080999; doi:10.1021/acs.inorgchem.6c00382)
Supplement: Supplementary file 1 [file ic6c00382_si_001.pdf]

Supporting Information

Crystal Structure of the High-Pressure Phase of $Ca(BH_4)_2$ and Further Structural Changes up to 70 GPa

Satoshi Nakano,1, Hiroshi Fujihisa,2 Hiroshi Yamawaki,2 Yuki Shibazaki,3*

Takumi Kikegawa,3 Shin-ichi Orimo4,5

[1]National Institute for Materials Science (NIMS), Tsukuba, Ibaraki 305-0044, Japan

[2]National Metrology Institute of Japan (NMIJ), National Institute of Advanced Industrial Science and Technology (AIST), Tsukuba, Ibaraki 305-8565, Japan

[3]Photon Factory (PF), Institute of Materials Structure Science (IMSS), High Energy Accelerator Research Organization (KEK), Tsukuba, Ibaraki 305-0801, Japan

[4]Advanced Institute for Materials Research (WPI-AIMR), Tohoku University, Katahira 2-1-1, Aoba-ku, Sendai, 980-8577, Japan

[5]Institute for Materials Research (IMR), Tohoku University, Katahira 2-1-1, Aoba-ku, Sendai, 980-8577, Japan

* Email: NAKANO.Satoshi@nims.go.jp

1. Rietveld refinement and structural model of the starting sample, α-$Ca(BH_4)_2$

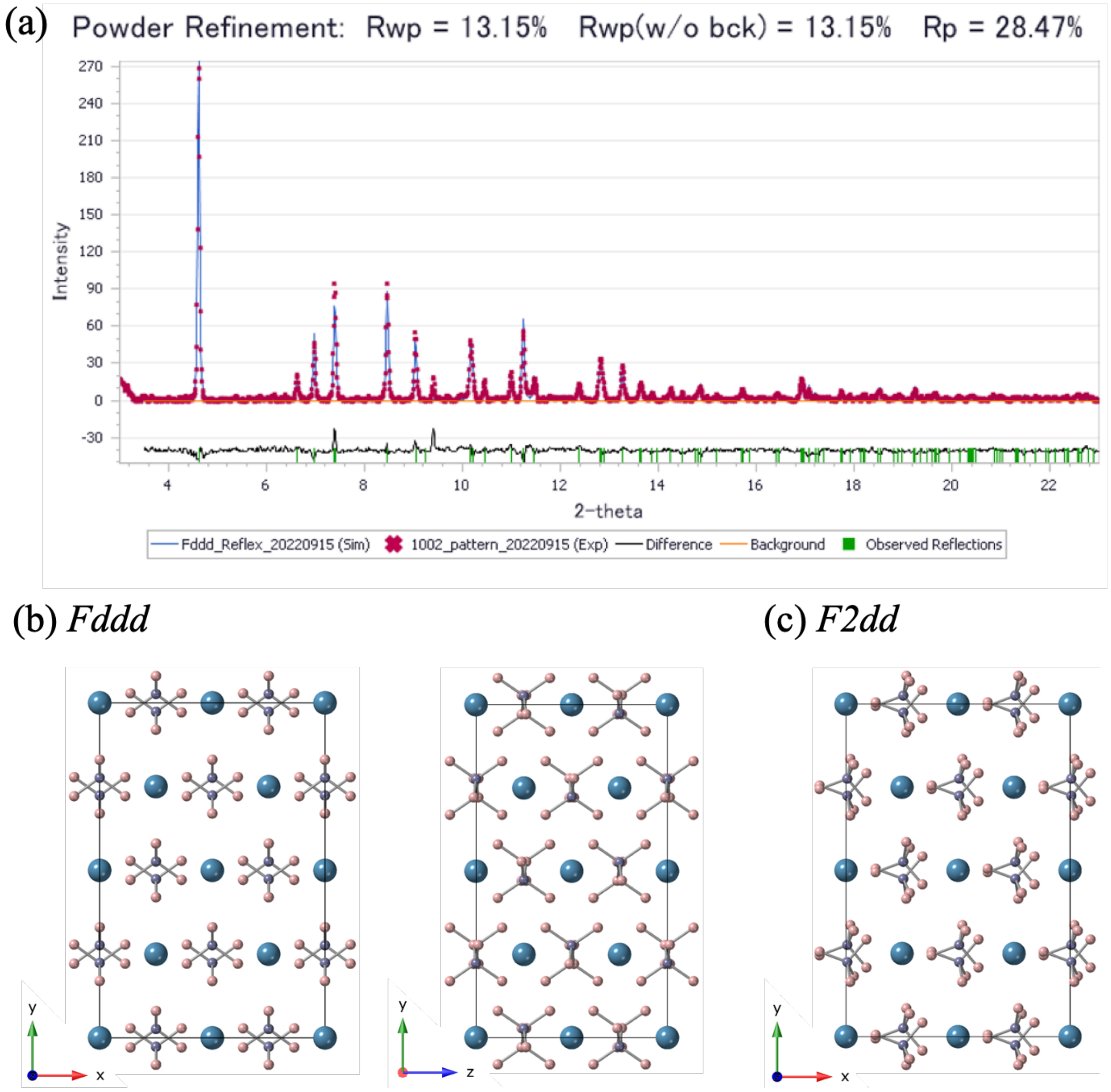

Figure S1. Results of the Rietveld refinement and the structural models of α-$Ca(BH_4)_2$. (a) The result of the Rietveld refinement for the X-ray diffraction (XRD) pattern obtained at 0.4 GPa and room temperature (RT), based on the *Fddd* structure. Red dots and blue lines represent the observed and calculated intensities, respectively. Vertical green bars indicate the positions of the calculated diffraction lines. The black line at the bottom shows the difference between the observed and calculated intensities. (b) The crystal structure of α-$Ca(BH_4)_2$ refined in the *Fddd* space group using density functional theory (DFT) calculations. The left and right panels show the *ab* and *bc* planes, respectively. Blue, purple, and pink spheres represent calcium, boron, and hydrogen atoms, respectively. (c) The *ab* plane of the crystal structure of α-$Ca(BH_4)_2$ reported by Filinchuk et al.[S1]

2. Comparison of the diffraction patterns of the second high-pressure state (HP2) with the structural model proposed by previous theoretical calculations

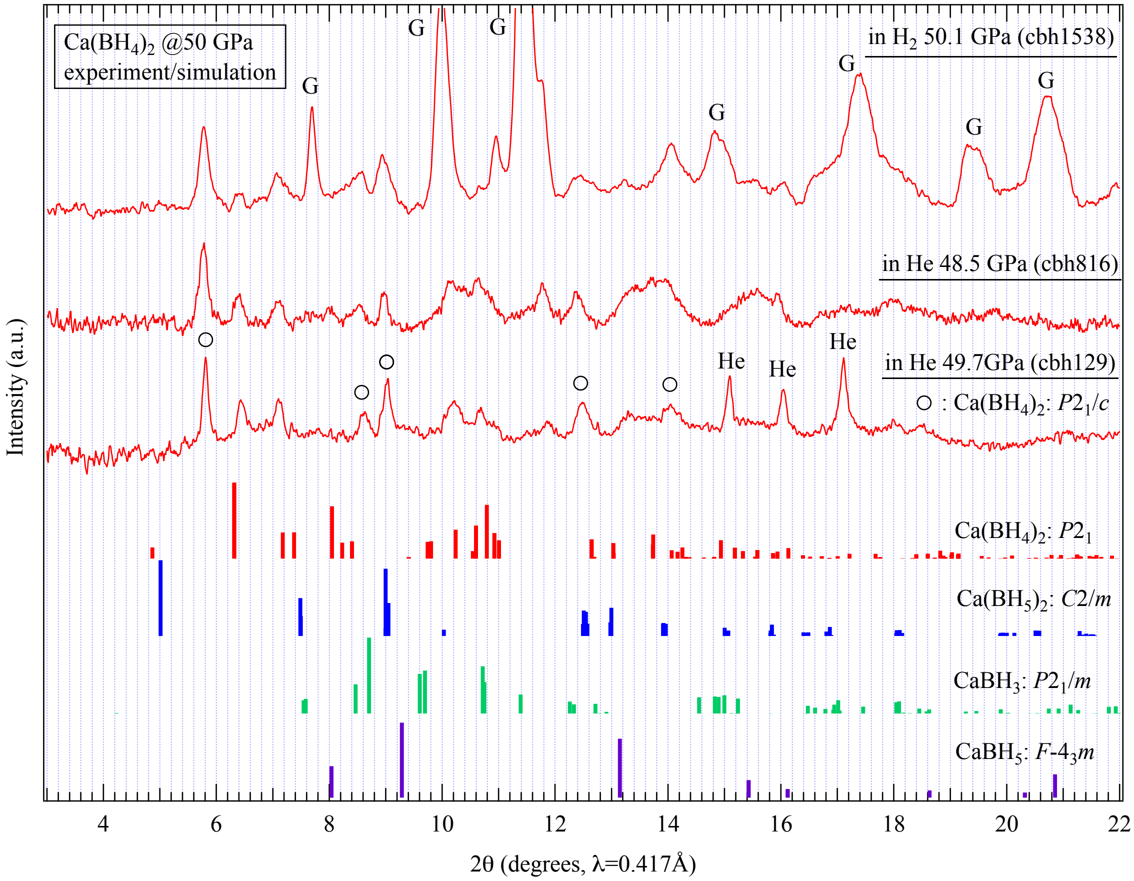

Figure S2. Comparison of the HP2 diffraction patterns obtained at approximately 50 GPa using helium and hydrogen pressure media with the structural models of the Ca–B–H compound proposed by Di Cataldo et al.[S2] Circles and "G" denote the diffraction peaks of the first high-pressure phase (HP1) and the rhenium gasket,[S3] respectively.

3. Hydrogenation behavior of a rhenium gasket in the experiment using hydrogen as the pressure medium

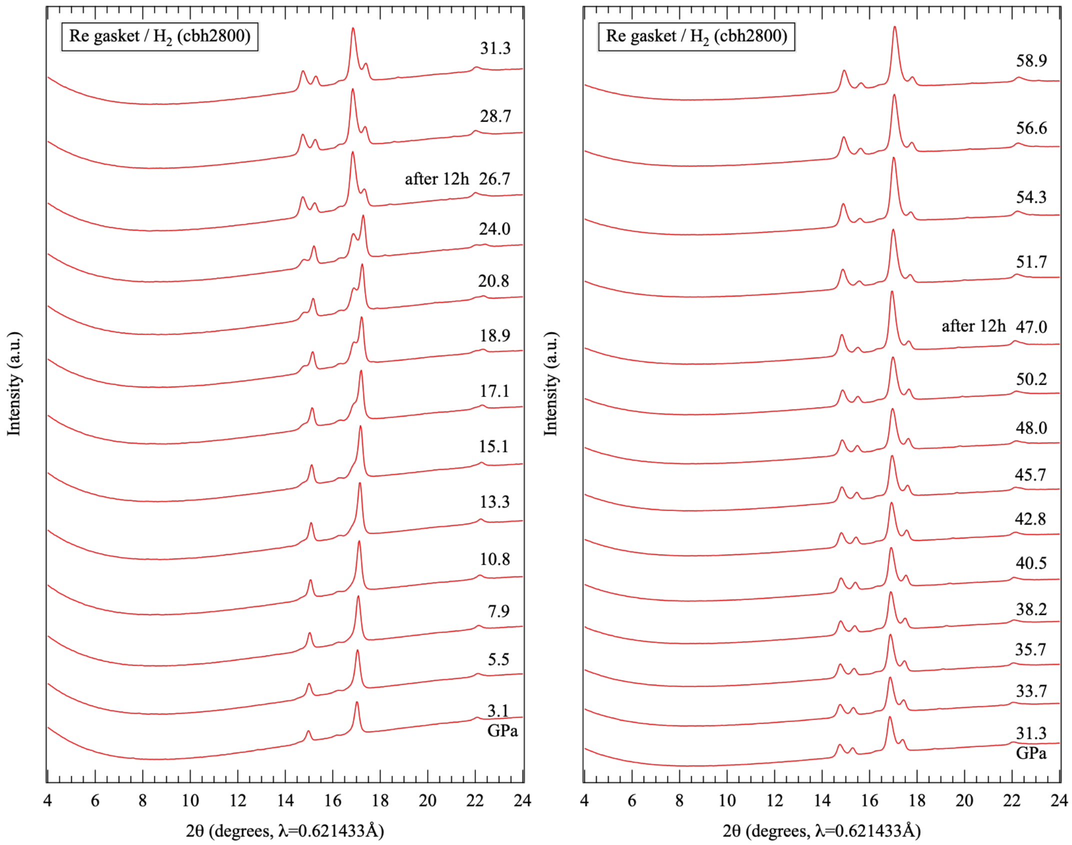

Figure S3. Pressure dependence of the XRD pattern of a rhenium gasket when the sample chamber was filled with hydrogen. The diffraction patterns were measured using a collimated X-ray beam with a diameter of 80 μm, which was slightly larger than the initial diameter of the sample chamber (60 μm). For diffraction patterns without a specific indication, 1 hour elapsed between measurements. At 3.1 GPa, shoulder peaks had appeared on the low-angle side of the rhenium peaks, and their intensity increased with increasing pressure. These shoulders correspond to rhenium hydride formed when hydrogen in the sample chamber penetrated the rhenium. The measurement at 26.7 GPa was performed approximately 12 hours after the measurement at 24.0 GPa, during which time the hydrogenation progressed significantly. This suggests that time significantly affects hydrogenation and that the rate of hydrogen diffusion is rate-limiting. The hydrogenation did not progress significantly above 50 GPa.

Scheler et al.[S4] determined the solubility of hydrogen in rhenium at high-pressure and high-temperature (HP/HT). When compared with the pressure dependence of rhenium hydride (ReH_x), the shift of the peak at 58.9 GPa in this experiment corresponds to x = 0.87. Therefore, the hydrogenated gasket is considered to be ε_2-$ReH_{0.87}$ with an NiAs-type structure.

4. Changes in the XRD pattern of HP2 during decompression

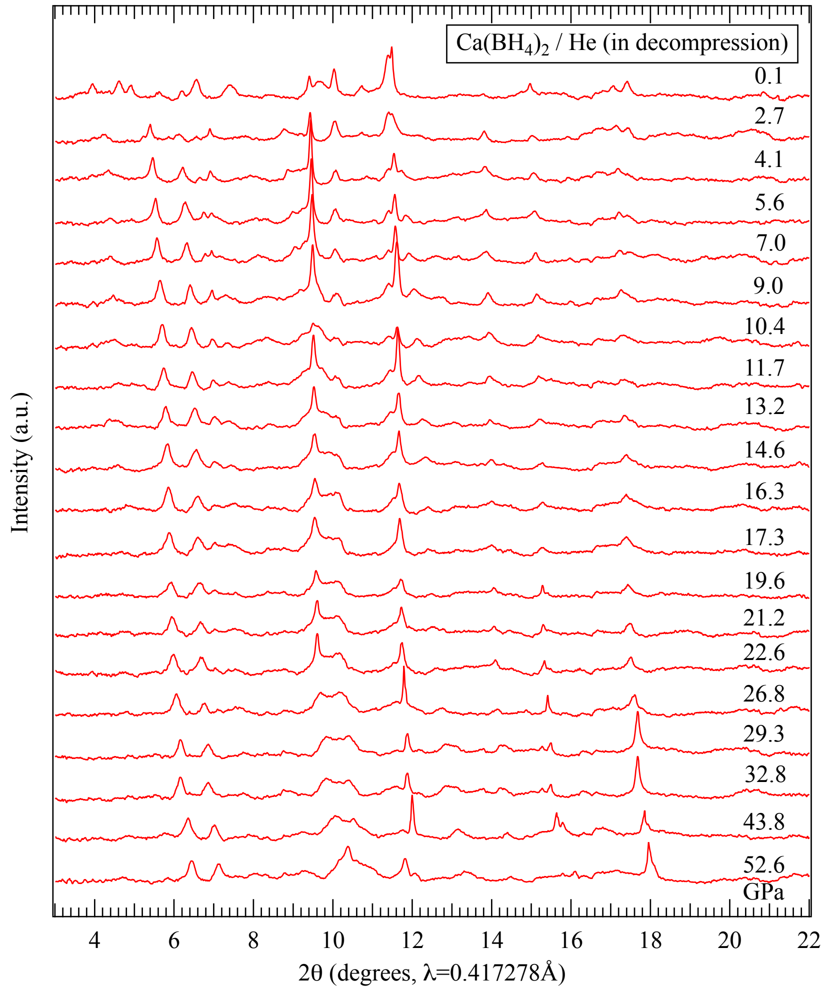

Figure S4. Changes in the XRD pattern of HP2 during decompression. No reverse transition from HP2 to HP1 or the ambient pressure (AP) phase occurred, and several characteristic peaks of HP2 were maintained at least up to approximately 2.7 GPa. Further decompression resulted in a change of the diffraction pattern near ambient pressure.

5. Comparison of the crystal structures of the α-$Ca(BH_4)_2$ and γ-$Ca(BH_4)_2$

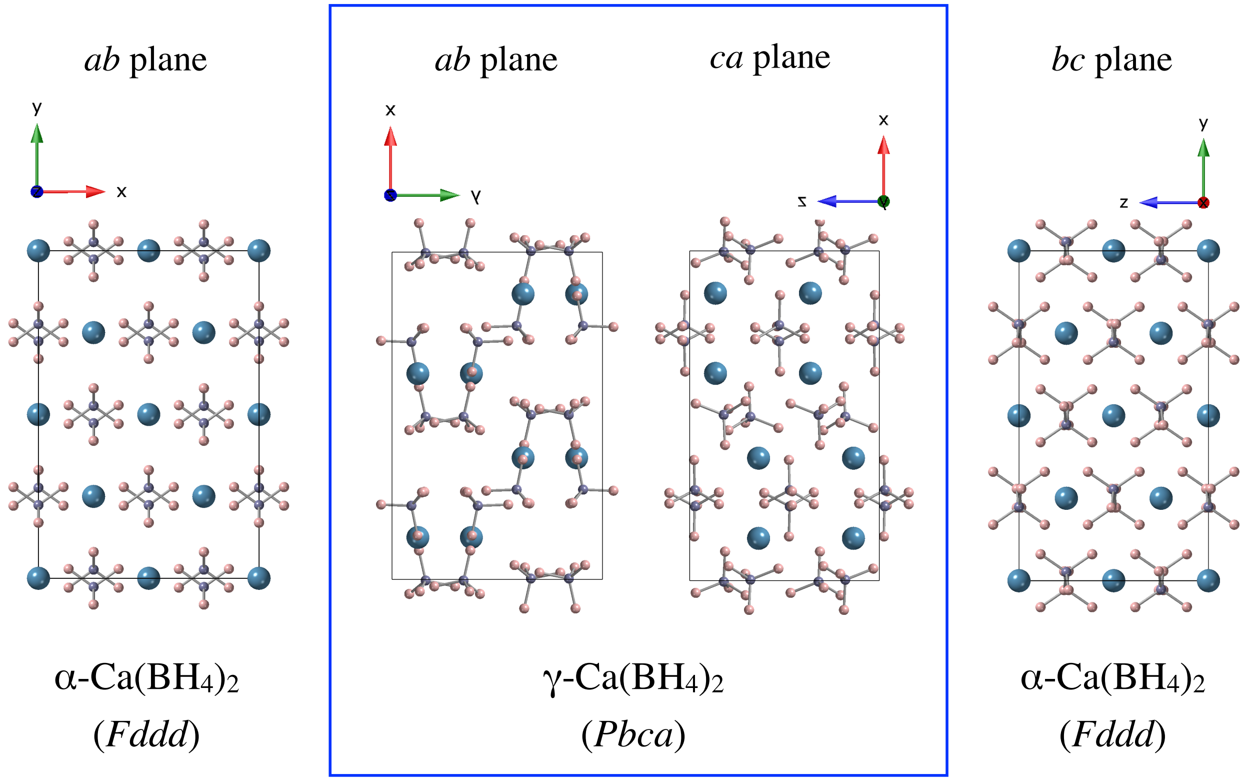

Figure S5. Comparison of the crystal structures of α-$Ca(BH_4)_2$ and γ-$Ca(BH_4)_2$. Both are orthorhombic crystals, and the comparison is based on the plane in which the number of ions in the unit cell is the same. The two central figures show the crystal structures of γ-$Ca(BH_4)_2$; the left figure shows the *ab* plane and the right figure shows the *ca* plane. On either side are the crystal structures of α-$Ca(BH_4)_2$, with the left-hand figure showing the *ab* plane and the right-hand figure showing the *bc* plane.

6. Raman scattering spectrum of the starting sample

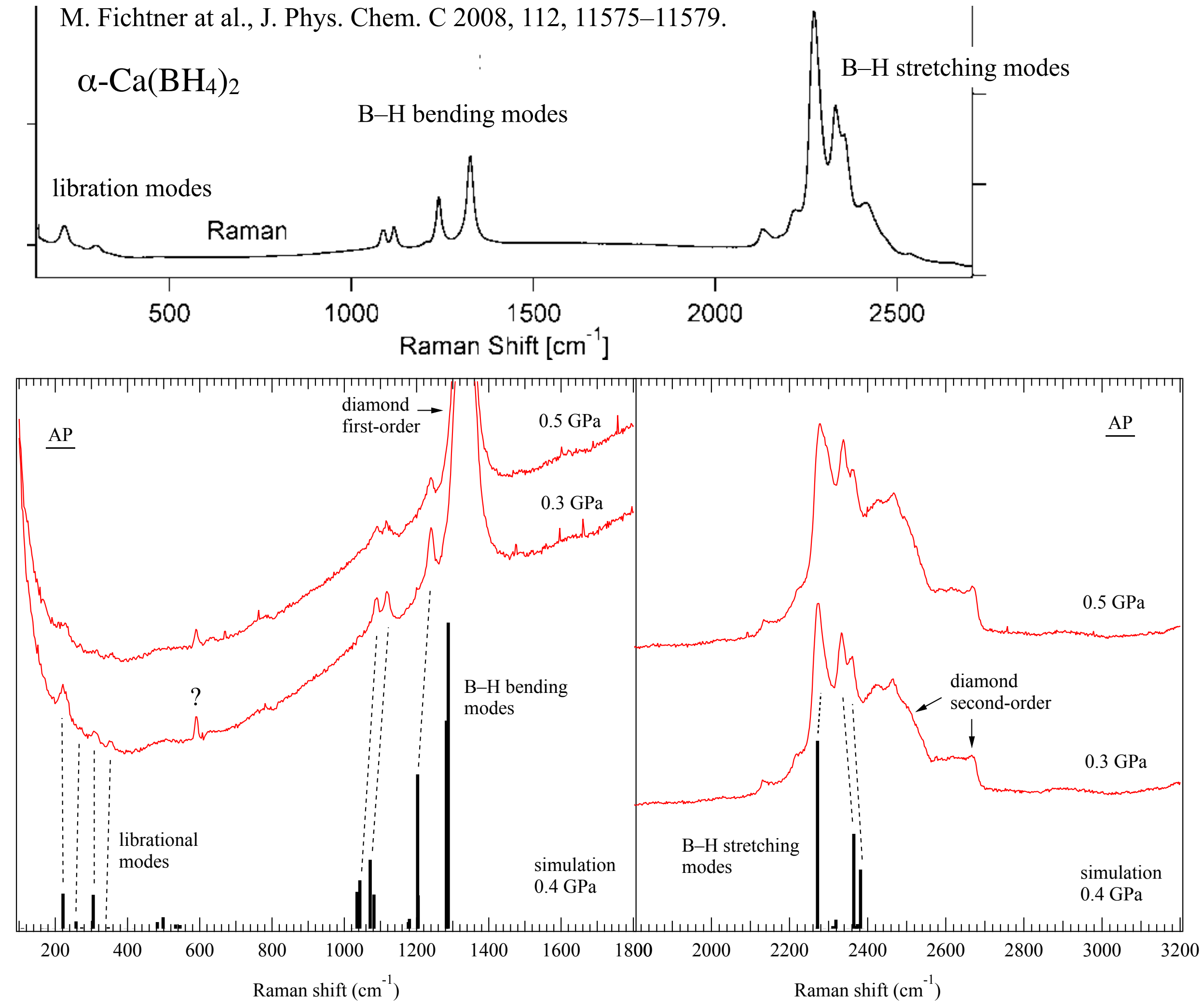

Figure S6. Raman spectra of the sample after loading helium gas as a pressure medium into the diamond anvil cell (DAC). Black vertical lines indicate the results of Raman peak simulations using DFT calculations. The spectra of the sample closely matche the previously reported Raman spectrum of α-$Ca(BH_4)_2$ by Fichtner et al.[S5] in all three mode regions: lattice and phonon modes (below 600 cm^{-1}), B–H bending modes (100–1500 cm^{-1}), and B–H stretching modes (2000–2600 cm^{-1}). A relatively sharp peak is also visible at approximately 590 cm^{-1}, but its origin is unclear and it may be due to a contamination. The simulation results reproduce the experimental spectra relatively accurately. However, the strongest peak in the B–H bending modes is obscured by the first-order peak of the diamond anvil. The other peaks are calculated to be lower in wavenumber by about 50 cm^{-1} in the simulation. Similar deviation has been noted in the comparison of experimental and theoretical values by Fichtner et al. In the B–H stretching modes, there is a slight discrepancy in wavenumber between the experimental and theoretical values, but the intensity ratios are in relatively good agreement.

7. Comparison of Raman scattering spectra of HP1 with simulation results

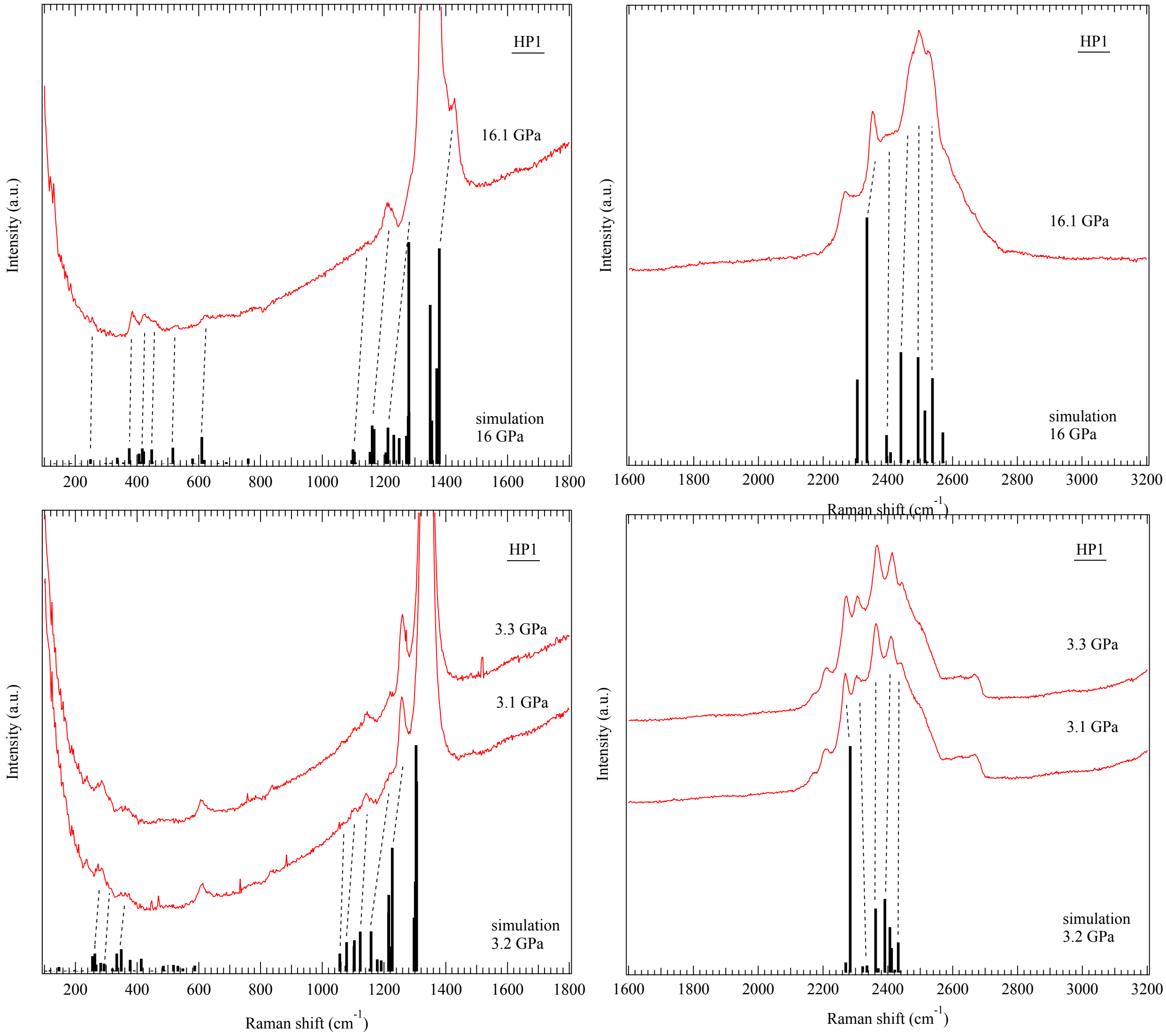

Figure S7. Comparison of Raman scattering spectra at approximately 3.2 GPa and 16 GPa with simulation results for HP1 ($P2_1/c$ structure). Although the Raman scattering spectra appear to be significantly different from each other, they both match the simulation results at each pressure, confirming that they are both HP1.

8. Comparison of Raman scattering spectrum at the initial stage of compression with spectra recovered from HP2 at near AP.

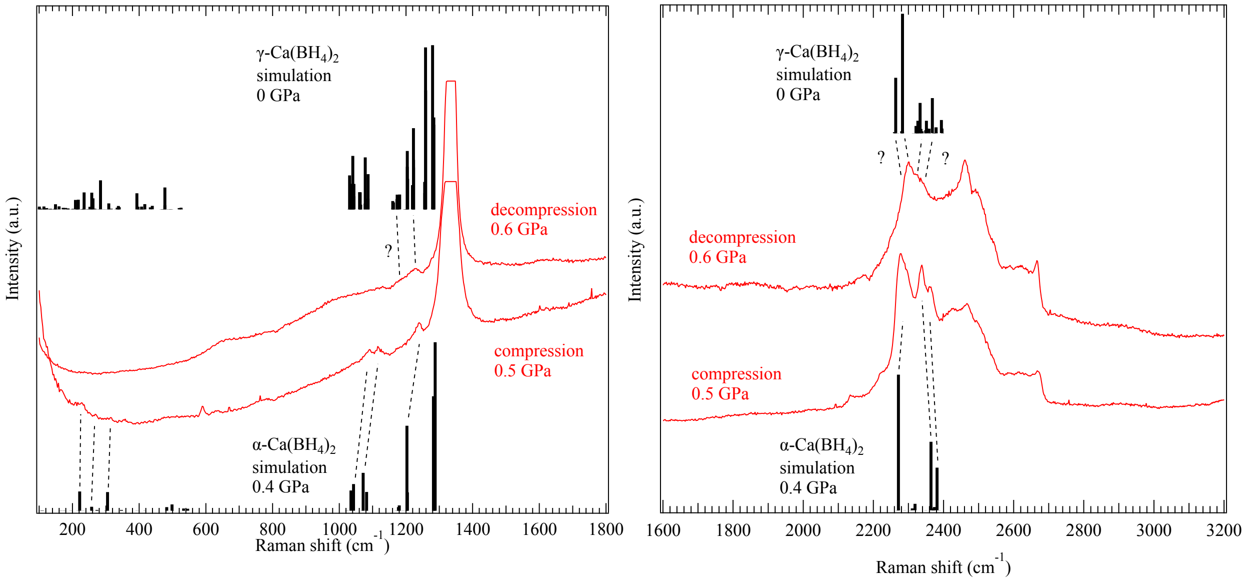

Figure S8. Spectra comparison at 0.5 GPa during compression and 0.6 GPa during decompression. The Black vertical lines demonstrate the results of DFT calculations of α-$Ca(BH_4)_2$ at 0.4 GPa and γ-$Ca(BH_4)_2$ at 0 GPa. The spectra recovered from HP2 differ from those of α-$Ca(BH_4)_2$. Lattice and librational modes are rarely observed, whereas the B–H bending and B–H stretching modes show spectra similar to those of γ-$Ca(BH_4)_2$.

9. HP/HT annealing of HP2

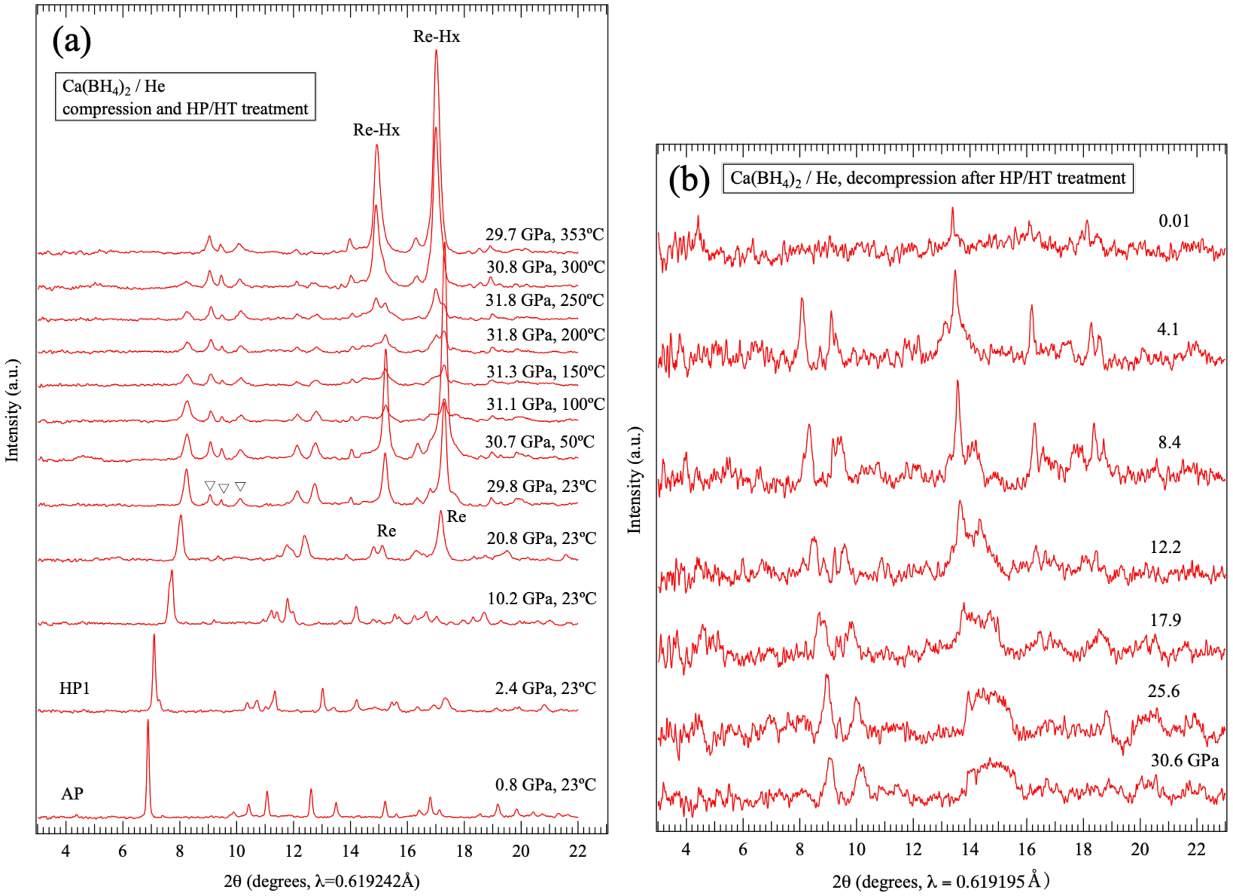

Figure S9. (a) Changes in the XRD pattern of a sample compressed to approximately 30 GPa within the HP2 pressure range and then heated to 353°C. (b) Changes in the XRD pattern during decompression at RT following HP/HT annealing.

The HP1 peak decreased with increasing temperature, but the HP2 peak did not appear to grow significantly. Therefore, annealing did not produce a high-quality HP2 diffraction pattern, and the HP2 structure could not be elucidated. Simultaneously measuring the diffraction pattern of the gasket, measured simultaneously showed a decrease in the rhenium peak above approximately 200°C, as well as the appearance of a new peak. This peak shift was not observed when the experiment was performed at RT using a helium pressure medium however it was observed when the experiment was performed at RT using a hydrogen pressure medium. This suggests that hydrogen is generated in the sample chamber as the temperature increases, resulting in the hydrogenation of the rhenium, i.e., the decomposition of the sample. Compared with the pressure dependence of ReH_x reported by Scheler et al.[S4], the peak shift in this experiment corresponds to $x = 0.65$, equivalent to ε2-$ReH_{0.65}$ of the NiAs-type structure. This indicates that at 30 GPa, HP1 decomposes above approximately 200 °C, releasing hydrogen, which reacts with the Re gasket to form $ReH_{0.65}$.

Kim et al. investigated the thermal decomposition of α-$Ca(BH_4)_2$ at AP and reported that a phase transition to the HT phase (β-$Ca(BH_4)_2$) occurred at approximately 167 °C, followed by a two-step decomposition reaction at 347 °C and 497 °C.[S6] However, their thermogravimetric analysis (TGA)

curve showed a decrease at approximately 220 °C, suggesting that some hydrogen desorption occurred during the phase transition to the β-phase. In this experiment, hydrogen absorption by Re was observed already at 200 °C and approximately 30 GPa. This may be due to the instability of HP1 at 30 GPa and the disordered structure during the phase transition to HP2, which may have caused the decomposition temperature to be lower than at AP.

Annealing caused the HP1 diffraction peak to disappear, but there was no decrease in the HP2 peaks. Therefore, it is possible that HP1 was decomposed during heating, leaving HP2 unaffected. However, this raises the question of whether the weak peaks observed in HP2 are an impurities or decomposition products, rather than another HP phase of $Ca(BH_4)_2$. During the decompression process, the peaks that appeared in HP2 remained down to 4.1 GPa, but disappeared when the pressure was reduced to 0.01 GPa, approaching AP.

References

(S1) Filinchuk, Y.; Rönnebro, E.; Chandra, D. Crystal structures and phase transformations in $Ca(BH_4)_2$. *Acta Materialia* **2009**, *57* (3), 732–738. DOI: 10.1016/j.actamat.2008.10.034

(S2) Di Cataldo, S.; von der Linden, W.; Boeri, L. Phase diagram and superconductivity of calcium borohyrides at extreme pressures. *Phys. Rev. B* **2020**, *102* (1), 014516. DOI: 10.1103/PhysRevB.102.014516

(S3) Anzellini, S.; Dewaele, A.; Occelli, F.; Loubeyre, P.; Mezouar, M. Equation of state of rhenium and application for ultra high pressure calibration. *J. Appl. Phys.* **2014**, *115* (4), 043511. DOI: 10.1063/1.4863300

(S4) Scheler, T; Degtyareva, O.; Gregoryanz, E. On the effects of high temperature and high pressure on the hydrogen solubility in rhenium Available. *J. Chem. Phys.* **2011**, *135* (21), 214501. DOI: 10.1063/1.3652863

(S5) Fichtner, M.; Chlopek, K.; Longhini, M.; Hagemann, H. Vibrational Spectra of $Ca(BH_4)_2$. *J. Phys. Chem. C* **2008**, *112* (30), 11575–11579. DOI: 10.1021/jp801482b

(S6) Kim, Y.; Reed, D.; Lee, Y.-S.; Lee, J. Y.; Shim, J.-H.; Book, D.; Cho, Y. W. Identification of the Dehydrogenated Product of $Ca(BH_4)_2$. *J. Phys. Chem. C* **2009**, *113* (14), 5865–5871, DOI: 10.1021/jp8094038
